# Efficacy and safety of pembrolizumab for the treatment of advanced or recurrent ovarian cancer: a meta-analysis based on single-arm studies

**DOI:** 10.3389/fimmu.2025.1662455

**Published:** 2025-10-01

**Authors:** Xiaodong Mi, Liming Tan, Tong Lin, Yimin Yang, Hongmei Liu, Fei Tuo

**Affiliations:** ^1^ Department of Obstetrics and Gynecology, People’s Hospital of Xiangxi Tujia and Miao Autonomous Prefecture, First Affiliated Hospital of Jishou University, Jishou, Hunan, China; ^2^ Department of Nursing, Medical College of Jishou University, Jishou, Hunan, China; ^3^ Department of Obstetrics and Gynecology, The Third Xiangya Hospital of Central South University, Changsha, Hunan, China; ^4^ Department of Obstetrics and Gynecology, Yongshun County People’s Hospital, Yongshun, Hunan, China

**Keywords:** pembrolizumab, ovarian cancer, PD-1, ICIS, immunotherapy

## Abstract

**Objective:**

Ovarian cancer is a common malignant tumor of the female reproductive system, and traditional treatments are unsatisfactory due to its intraperitoneal spreading mechanism and its biologic characteristic of being prone to drug resistance. Pembrolizumab has demonstrated high objective remission and overall survival rates in a variety of solid tumors, and clinical trials are now available to explore its efficacy in the treatment of ovarian cancer. The objective of this systematic review and meta-analysis was to synthesize the findings of multiple clinical studies in order to evaluate the effectiveness and safety of the immunotherapeutic agent Pembrolizumab in patients diagnosed with advanced or recurrent ovarian cancer.

**Methods:**

A comprehensive search of literature published up to 19 November 2024 was conducted in the following databases: PubMed, Embase, Cochrane Library, Web of Science, Ovid_medline, Scopus, and ProQuest. The outcomes related to the administration of pembrolizumab in patients with advanced or recurrent ovarian cancer were extracted, including objective remission rate (ORR), disease control rate (DCR), overall survival (OS), progression-free survival (PFS), and treatment-related adverse events (AEs). A further meta-analysis was then performed.

**Results:**

This meta-analysis comprised 625 patients across nine studies. A total of 617 patients were involved in the efficacy assessment, while 592 patients were included in the safety assessment. The pooled analysis indicated an ORR of 24% (95% confidence interval [CI], 0.13–0.35), a DCR of 63% (95% CI, 0.49–0.77), a median progression-free survival (mPFS) of 4.82 months (95% CI, 3.29–6.35), and a median overall survival (mOS) of 13.54 months (95% CI, 10.35–16.73). Subgroup analysis indicated that the ORR in the PD-L1-positive group was 24% (95% CI, 0.12–0.36), while the ORR in the PD-L1-negative group was 18% (95% CI, 0.09–0.27). No statistically significant difference was observed between the two groups. The ORR was 26% (95% CI, 0.13–0.33) for patients administered a 200 mg dose every three weeks (q3w), while it was 12% (95% CI, 0.02–0.30) for those receiving a 10 mg/kg dose every two weeks (q2w). The overall incidence of adverse reactions of any grade was 81% (95% CI, 0.71–0.91), whereas the incidence of grade 3 or higher adverse reactions was 32% (95% CI, 0.09–0.54).

**Conclusions:**

This meta-analysis indicates that pembrolizumab treatment for patients with advanced or recurrent ovarian cancer exhibits significant efficacy and an acceptable safety profile.

**Systematic review registration:**

https://www.crd.york.ac.uk/PROSPERO/view/ identifier CRD42024620116.

## 1 Introduction

Ovarian cancer (OC) is the third most common and deadly malignancy of the female reproductive system. In 2022, the global burden of OC is estimated to be approximately 325,000 new cases and approximately 207,000 deaths, with a projected 47 percent increase in the incidence of OC and a 58 percent increase in deaths by 2045 (1). Northern Europe and North America have historically experienced the highest incidence rates of OC; however, recent trends indicate a shift in these regions, with a decline in incidence. Conversely, there has been an increase in the incidence of OC in certain regions of Eastern Europe and Asia, particularly among women under the age of 50 (2). OC typically manifests insidiously, with patients often exhibiting no specific symptoms in the early stage. However, approximately 70% of patients have already reached the advanced stage with distant metastases at the time of diagnosis (3). The prognosis for advanced OC is poor, with a 5-year survival rate of approximately 25 percent and a 10-year survival rate of approximately 15 percent (4). The standard first-line treatment options for OC include surgery or neoadjuvant chemotherapy followed by surgery (5), followed by platinum-containing chemotherapy. Approximately 75-80% of patients with OC respond to these initial treatments, but 70% of patients eventually experience recurrence, develop drug resistance, and die from their cancer (6, 7). These findings underscore the imperative for the development of novel therapeutic interventions for OC, with particular emphasis on novel therapeutic strategies for recurrent or advanced OC.

Immune checkpoint inhibitors (ICIs) are a category of pharmaceuticals that target immunological receptors on T cells, therefore augmenting anti-tumor immune responses (8). Unlike traditional treatment methods, ICI therapy has demonstrated the ability to rejuvenate immune system activity, allowing the body to fight cancer cells (9). In specific cases, these medicines have shown extended effectiveness and low toxicity (10). Following the approval of ipilimumab in the United States in 2011, ICIs have advanced considerably in cancer immunotherapy (11). The FDA-approved immune checkpoint inhibitors (ICIs) currently encompass several categories: anti-PD-1 inhibitors, anti-PD-L1 inhibitors, anti-CTLA-4 inhibitors, and anti-LAG-3 inhibitors. New immune checkpoint targets, including TIGIT, TIM-3, VISTA, and CD3L1, are presently undergoing clinical development (12). In the past decade, PD-1, PD-L1, and CTLA-4 antibodies have emerged as the predominant agents in immunotherapy (13, 14). Pembrolizumab, introduced in 2014 as the first PD-1 inhibitor, initiated a new era in cancer immunotherapy (15). Since that time, several inhibitors aimed at PD-1 and its ligand PD-L1, including nivolumab, atezolizumab, durvalumab, and avelumab, have received approval from the U.S. Food and Drug Administration (FDA) for the treatment of various malignant tumors (16, 17).

Pembrolizumab is a specific humanized IgG4 monoclonal antibody that effectively inhibits the interaction between the programmed cell death protein PD-1 on T cells and its ligand, consequently activating T cell-mediated antitumor immune responses (18, 19). Pembrolizumab has received approval from the FDA for the treatment of certain tumor types, including melanoma, non-small cell lung cancer, head and neck squamous cell carcinoma, and renal cell carcinoma (20). Treatment options consist of pembrolizumab administered either as monotherapy or in conjunction with chemotherapy or radiotherapy. The KEYNOTE-716 trial demonstrated that postoperative adjuvant pembrolizumab monotherapy significantly enhanced survival rates in patients with stage IIB or IIC melanoma without distant metastasis, consistently decreased recurrence risk, and exhibited a predictable safety profile (21). The KEYNOTE-189 trial demonstrated that the combination of pembrolizumab and chemotherapy significantly extended overall survival and progression-free survival compared to chemotherapy alone in previously untreated metastatic non-squamous NSCLC lacking EGFR/ALK mutations (22). The KEYNOTE-040 trials indicate that pembrolizumab significantly extends overall survival in patients with recurrent or metastatic squamous cell carcinoma of the head and neck who have progressed following platinum-based therapy, in comparison to standard treatments like methotrexate, docetaxel, or cetuximab, while also demonstrating superior safety (23). The KEYNOTE-564 trial indicated that pembrolizumab, when used as adjuvant therapy following nephrectomy in patients with renal cell carcinoma, significantly enhanced disease-free survival compared to placebo and exhibited a favorable safety profile (24). In addition to the use of common solid tumors, a single-arm, open-label phase II basket trial conducted by the MD Anderson Cancer Center in the United States employed pembrolizumab in patients diagnosed with advanced rare cancers (alveolar soft tissue sarcoma, testicular mesothelioma, intracranial meningioma, and neuroblastoma) who had demonstrated disease progression following standard treatment modalities. The results demonstrated that a subset of patients exhibited a certain degree of efficacy and were well tolerated (25).

A plethora of clinical trials have been conducted to ascertain the efficacy and safety of pembrolizumab in the treatment of OC. However, the majority of these clinical trials are single-arm, thus lacking high-quality randomized controlled trials (RCTs). In this paper, all eligible studies will be included to comprehensively analyze the efficacy and safety of pembrolizumab in the treatment of advanced or recurrent OC, in anticipation of providing clinicians with more accurate data and guidance when choosing treatment options.

## 2 Materials and methods

This meta-analysis was conducted in accordance with the Preferred Reporting Items for Systematic Reviews and Meta-Analysis (PRISMA) (26) guidelines and has been registered with PROSPERO (NO: CRD42024620116).

### 2.1 Search strategy

A comprehensive search of the extant literature was conducted, encompassing publications up to November 18, 2024, in the following databases: PubMed, Embase, Web of Science, Cochrane Library, Ovid_medline, Scopus, and ProQuest. The meta-analysis did not impose any language restrictions. The subject terms employed in the PubMed database were “Ovarian Neoplasms” [Mesh] and “pembrolizumab” [**Supplementary Concept**]. The detailed search strategy can be seen in **Table 1**.

**Table 1:** PubMed searching strategy.

Number	Query	Results
#7	#3 AND #6	**50**
#6	#4 OR #5	**4,722**
#5	Search: (((MK-3475[Title/Abstract]) OR (Keytruda[Title/Abstract])) OR (lambrolizumab[Title/Abstract])) OR (SCH-900475[Title/Abstract]) Sort by: Most Recent	**241**
#4	Search: “pembrolizumab” [**Supplementary Concept**] Sort by: Most Recent	**4,583**
#3	#2 OR #1	**124,209**
#2	Search: ((((((((((((((((Neoplasm, Ovarian[Title/Abstract]) OR (Ovarian Neoplasm[Title/Abstract])) OR (Neoplasms, Ovarian[Title/Abstract])) OR (Ovary Neoplasms[Title/Abstract])) OR (Neoplasm, Ovary[Title/Abstract])) OR (Neoplasms, Ovary[Title/Abstract])) OR (Ovary Neoplasm[Title/Abstract])) OR (Ovary Cancer[Title/Abstract])) OR (Cancer, Ovary[Title/Abstract])) OR (Cancers, Ovary[Title/Abstract])) OR (Ovary Cancers[Title/Abstract])) OR (Cancer of Ovary[Title/Abstract])) OR (Cancer of the Ovary[Title/Abstract])) OR (Ovarian Cancer[Title/Abstract])) OR (Cancer, Ovarian[Title/Abstract])) OR (Cancers, Ovarian[Title/Abstract])) OR (Ovarian Cancers[Title/Abstract]) Sort by: Most Recent	**77,287**
#1	Search: “Ovarian Neoplasms”[Mesh] Sort by: Most Recent	**99,746**

Significance of Bold Values in **Table 1**: Using '[**Supplementary File**]' in PubMed literature searches ensures precise matching of drug names and prevents result omissions due to synonyms or spelling variations.

### 2.2 Inclusion and exclusion criteria

The inclusion criteria were as follows (1): The study population was patients with a pathologically confirmed diagnosis of OC, with advanced or recurrent disease confirmed by pathology or imaging; (2) The intervention was pembrolizumab monotherapy or pembrolizumab in combination with other treatments. (3) The types of studies were RCTs, single-arm trials, prospective cross-sectional cohort studies. The following types of studies were excluded from the present review: case reports, *in vitro* experiments, reviews, abstracts, letters, retrospective studies, and pathological studies. In instances where authors have published multiple studies utilizing the same dataset, the most recent or comprehensive study was selected for inclusion. Studies that were duplicates or included other tumors for which data could not be extracted separately were excluded from the analysis. The titles and abstracts of all retrieved studies were then screened according to the search strategy, and studies that did not meet the inclusion criteria were excluded. A comprehensive extraction of information and data was conducted from studies that met the predetermined inclusion criteria. The information and data to be extracted are outlined below: authors’ names, affiliations, year of publication, study type, number of cases, patient age, dose of pembrolizumab, combination with other therapies, and outcome parameters.

### 2.3 Study selection strategies

Researchers Xiaodong Mi and Fei Tuo completed the preliminary literature screening by methodically reviewing titles and abstracts based on pre-established inclusion/exclusion criteria, then proceeded to conduct a further screening through detailed full-text reading, during which any controversial literature was resolved through consultation and final decisions made with a third researcher, Tong Lin.

### 2.4 Data extraction

The baseline characteristics of the patients collected for this study encompassed the following variables: age, number of cases, number of previous lines of chemotherapy received, tumor pathology type, platinum resistance status, BRCA gene mutation status, homologous recombination repair defect (HRD) status, programmed death receptor-1/programmed death ligand-1 (PD-1/PD-L1) expression status, dose of pembrolizumab administered, and specific information on combination therapy regimens. The efficacy assessment indexes comprised the following: ORR and DCR. Furthermore, the ORR and DCR were also considered. In addition to these, OS and PFS were extracted as indicators of long-term efficacy. The safety assessment was based on the occurrence of AEs, which were categorized as follows: firstly, the incidence of all adverse events; secondly, the incidence of grade 1–2 AEs; and thirdly, the incidence of grade 3–4 AEs. AEs encompassed include, but are not limited to, anemia, neutropenia, thrombocytopenia, malaise, abnormal liver function, pain, hypertension, hand-foot syndrome, rash, diarrhea, loss of appetite, weight loss, oral mucositis, thyroid dysfunction, and proteinuria.

### 2.5 Quality assessment

The methodological quality of the included literature was assessed using the Risk of Bias in Non-randomized Studies of Interventions (ROBINS-I) tool, which covers seven core assessment domains, each of which consists of structured questions with response options including ‘yes,’ ‘probably,’ ‘probably not,’ ‘no,’ and ‘no information.’ Response options include “yes,” “probably,” “probably not,” “no,” and “no information.” The final risk of bias was then determined as “low risk,” “moderate risk,” “serious risk,” or “borderline risk” based on the comprehensive assessment of the questions in each domain. The research team has completed the risk assessment for all seven domains (27, 28). To ensure the objectivity and reliability of the assessment, Xiaodong Mi and Fei Tuo independently evaluated the quality of all the included literature using the ROBINS-I scale and quantitatively analysed the inter-assessor agreement using the weighted Cohen’s kappa coefficient (κ) (29).

### 2.6 Statistical analysis

The statistical analysis of the study data was conducted using R version 4.4.1. The primary endpoints encompassed efficacy and safety assessments, while secondary endpoints involved survival analyses. The analysis of single-arm rates was performed using generalized linear mixed models (GLMM), calculated by metaprop functions, and interval estimation was conducted using the Clopper-Pearson method. The calculation of combined analyses of single-arm continuous variables was performed using the metamean function. Statistical heterogeneity was assessed using the I^2^ statistic and Cochran’s Q test. Given that I^2^ values in single-arm meta-analyses usually exceeded 90%, this study uniformly used a random-effects model for the combined analyses rather than choosing a fixed-effects or random-effects model based on the level of heterogeneity to ensure the robustness and reliability of the results (30). The results of the combined studies were further illustrated using forest plots, and the Egger test was employed to evaluate publication bias. A p-value of less than 0.05 signifies a substantial publishing bias.

## 3 Results

### 3.1 Literature search

**Figure 1:**
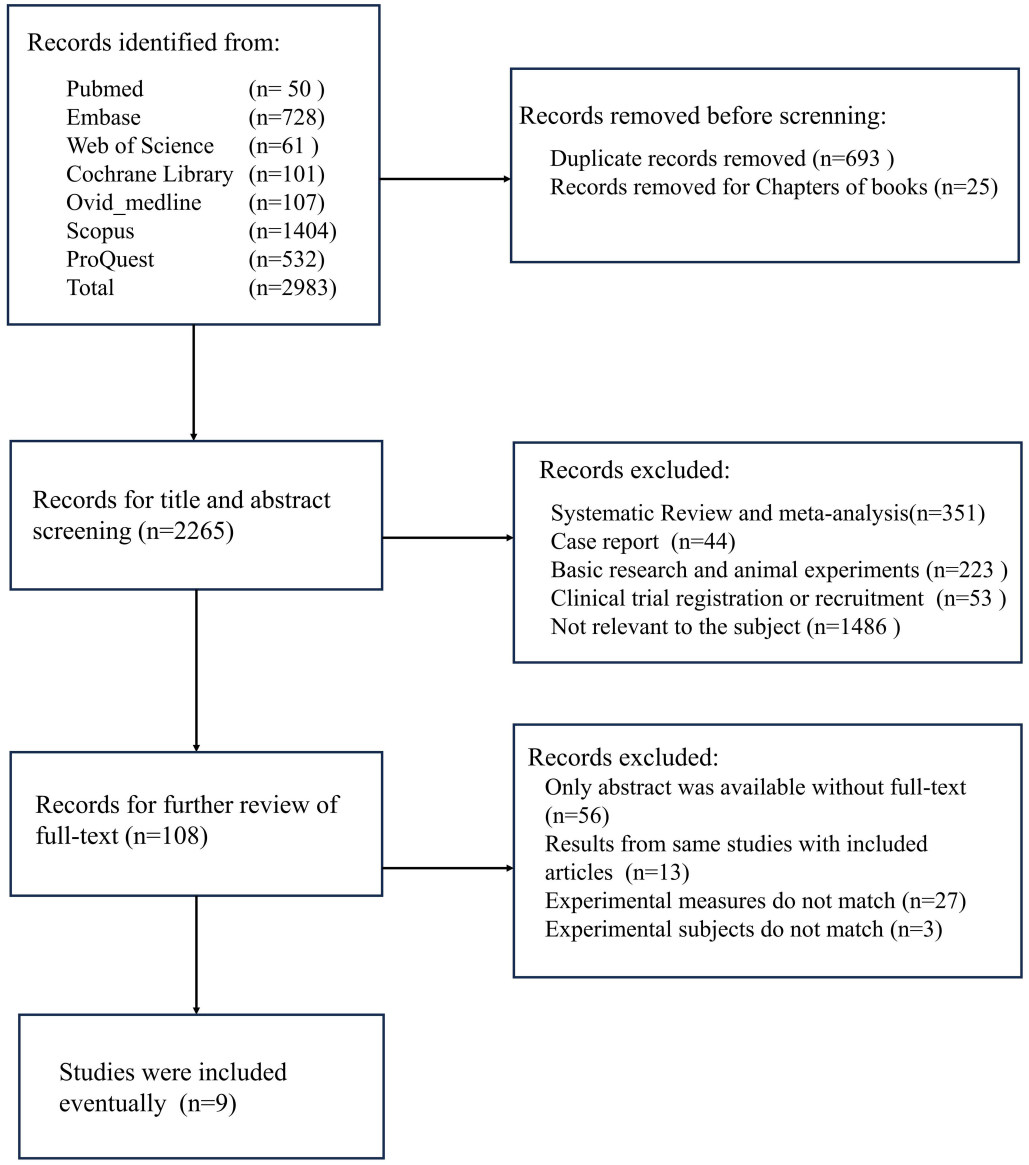
Research screening flowchart.

A comprehensive search was conducted for this study, encompassing seven major databases, which resulted in a total of 2,938 documents being reviewed. Following the exclusion of duplicate studies and book chapters, the number of documents was reduced to 2,265. Following a thorough examination of the titles and abstracts, a total of 351 systematic reviews and meta-analyses, 44 case reports, 223 basic research papers, 53 clinical trial recruitment or registration information, and 1,486 papers unrelated to the research topic were excluded. Following an exhaustive evaluation of the full texts, 56 studies were excluded due to the unavailability of full text, 13 studies were excluded as they were duplicate reports, 27 studies were excluded for failing to correspond with the study’s theme, and 3 studies were excluded due to inconsistencies in primary outcome measures, rendering meta-analysis unfeasible. A total of 9 studies were incorporated into this meta-analysis (**Figure 1**).

### 3.2 Baseline characteristics

This meta-analysis included nine studies conducted in the United States, Greece, China, France, and Canada. All studies were prospective trials, comprising eight single-arm clinical trials and one non-randomized controlled trial. Due to the advanced recurrent OC present in all patients of this non-randomized controlled trial (31) and the uniformity of the intervention, the two cohorts from this study were amalgamated to satisfy the inclusion criteria for this meta-analysis. This meta-analysis comprised 625 patients in total. The efficacy assessment dataset comprised an effective sample size of 617 patients. Eight patients were excluded from the efficacy analysis due to the following reasons: In the study conducted by Panagiotis A K (32), two patients withdrew from the trial during treatment for personal reasons. In Elizabeth K Lee’s study (33), three patients discontinued the trial due to disease progression or severe allergic reactions to chemotherapy drugs after the initial treatment, resulting in incomplete efficacy follow-up. In Christine’s study (34), three patients withdrew due to complications associated with OC recurrence, preventing them from completing subsequent imaging assessments. The total number of patients eligible for meta-analysis in safety assessment datasets was 592. Panagiotis’s study (32) encompassed Phase I and Phase II cohorts, with safety data for 14 patients in the Phase I cohort deemed incomplete and therefore excluded from the meta-analysis. Safety information was fully extracted for 53 OC patients in the Phase II cohort. John B Lee’s study (35) employed a distinct method for calculating the incidence of AEs, which precluded a meta-analysis of effect sizes with the other included studies. Christine’s study (34) categorized AEs statistics into the combination therapy phase and the single-agent maintenance phase, revealing overlap between the two phases. To prevent data duplication, only the incidence of AEs from the single-agent maintenance phase was included.

Aggregate analysis of baseline characteristics across included studies determined that patient age ranged from 25 to 89 years. The majority of patients exhibited an Eastern Cooperative Oncology Group (ECOG) performance status of 0 (n=367) or 1 (n=194). Serous carcinoma represented the predominant histological subtype (n=425). Evaluation of programmed death-ligand 1 (PD-L1) expression status demonstrated that 341 patients tested positive, while 210 tested negative. Baseline characteristics of all enrolled patients are summarized in **Table 2**, with study-specific features detailed in **Table 3**.

**Table 2:** Patient characteristics at baseline.

Characteristic	N
All patient	625
ECOG performance status	
0	367
1	194
Histology	
serous	425
Clear cell carcinoma	39
Endometrioid carcinoma	31
Prior bevacizumab	109
Prior PARP inhibitor	35
Platinum status	
Platinum-sensitive	17
Platinum-resistant	153
Platinum-refractory	24
PD-L1 status	
Positive	341
Negative	210
Number of previous lines of therapy	
1	101
2	152
3	129
4	49
≥5	60

The data presented in this table is limited due to incomplete information on all enrolled patients, with some entries missing. Only the core statistical results of each study are summarized.

**Table 3:** Basic information on the studies.

Study year	Registration number	Nation	Trial phase	Intervention	Pembrolizumab usage and dosage	Sample size	Age, median (range)	Patients	Endpoints
Panagiotis A K2019 (32)	NCT02657889	USA	Single-Arm Phases 1 and 2 Trial	Pembrolizumab + Niraparib	200mg iv q3w	62	60(46 - 83)	Recurrent Platinum-Resistant Ovarian Carcinoma	①②④⑤
U.A. Matulonis 2019 (31)	KEYNOTE-100 NCT02674061	Multinational	phase 2, open-label, multi-center study	Pembrolizumab	200mg iv q3w	376	61 (25-89)	Advanced Recurrent Ovarian Cancer	①②⑤
Andrea Varga 2019 (47)	KEYNOTE-028 NCT02054806	USA, China, French	Single-Arm, phase Ib trial	Pembrolizumab	10mg/kg iv q2w	26	57.5 (44–75)	PD-L1 positive advanced ovarian cancer	①②③④⑤
Elizabeth K Lee 2020 (33)	NCT02865811	USA	Single-Arm, phase 2 trial	Pembrolizumab + PLD	200mg iv q3w	26	60(28.3-79)	Platinum-Resistant Ovarian Carcinoma	①②③④⑤
Christine S W 2021 (34)	NCT02608684	USA	a single-center, open-label, Single-arm phase II trial	Pembrolizumab + gemcitabine and cisplatin	200mg iv q3w	21	55(46-71)	Recurrent platinum-resistant ovarian cancer.	①②③④⑤
John B Liao 2021 (35)	NCT03029598	USA, Canada	Phase I/II single arm trial	Pembrolizumab + carboplatin	200mg iv q3w	29	65(41-80)	recurrent platinum-resistant ovarian, fallopian tube, and primary peritoneal cancer	①②③④⑤
Zsiros E 2021 (48)	NCT02853318	USA	open-label, single-arm phase 2 cohort study	Pembrolizumab + Bevacizumab +Cyclophosphamide	200mg iv q3w	40	62(45-89)	recurrent fallopian tube, and primary peritoneal cancer	①②③④⑤
Lilian T Gien 2024 (49)	NCT02853318	USA	Single arm, two-stage, phase 2 trial	Pembrolizumab + epacadostat	200mg iv q3w	14	65(44-89)	recurrent clear cell carcinoma of the ovary	①②③④⑤
Antonio G 2024 (36)	LEAP - 005 NCT03797326	Multinational	phase 2, multicenter, multicohort, open-label study	Pembrolizumab + Lenvatinib	200mg iv q3w	31	62(40-76)	advanced ovarian cancer	①②③④⑤

①ORR; ②DCR; ③mPFS; ④mOS; ⑤AEs;

### 3.3 Quality assessment

Two researchers assessed the risk of bias of 9 studies based on the ROBINS-I tool, and the Cohen’s kappa coefficient of overall bias was 0.667, which suggests that the consistency of the risk of bias assessment of the literature between the two researchers is high. To address the inconsistency of the assessment results between the two researchers, we introduced a third researcher, Tong Lin, to jointly assess the results. **Table 4** displays the final results. One study was deemed to be at serious risk of bias, four at moderate risk, and four at low risk.

**Table 4:** Quality assessment of the non-randomized controlled studies (ROBINS-I).

Study	Bias due to confounding	Bias in selection of participants into the study	Bias in classification of interventions	Bias due to deviations from intended interventions	Bias due to missing data	Bias in measurement of outcomes	Bias in selection of the reported result	Overall bias
Panagiotis A K 2019 (32)	Serious	Low	Low	Moderate	Low	Moderate	Low	Serious
U.A. Matulonis 2019 (31)	Low	Low	Low	Low	Low	Low	Low	Low
Andrea Varga 2019 (47)	Serious	Low	Low	Low	Low	Moderate	Low	Moderate
Elizabeth K Lee 2020 (33)	Moderate	Moderate	Low	Low	Moderate	Low	Low	Moderate
Christine S W 2021 (34)	Low	Low	Moderate	Moderate	Low	Low	Low	Moderate
John B Liao 2021 (35)	Serious	Low	Low	Low	Low	Low	Low	Low
Zsiros E 2021 (48)	Moderate	Low	Low	Low	Low	Low	Low	Low
Lilian T Gien 2024 (49)	Moderate	Low	Low	Low	Low	Low	Low	Low
Antonio G 2024 (36)	Moderate	Low	Low	Moderate	Low	Low	Low	Moderate

### 3.4 Tumor response

**Figure 2:**
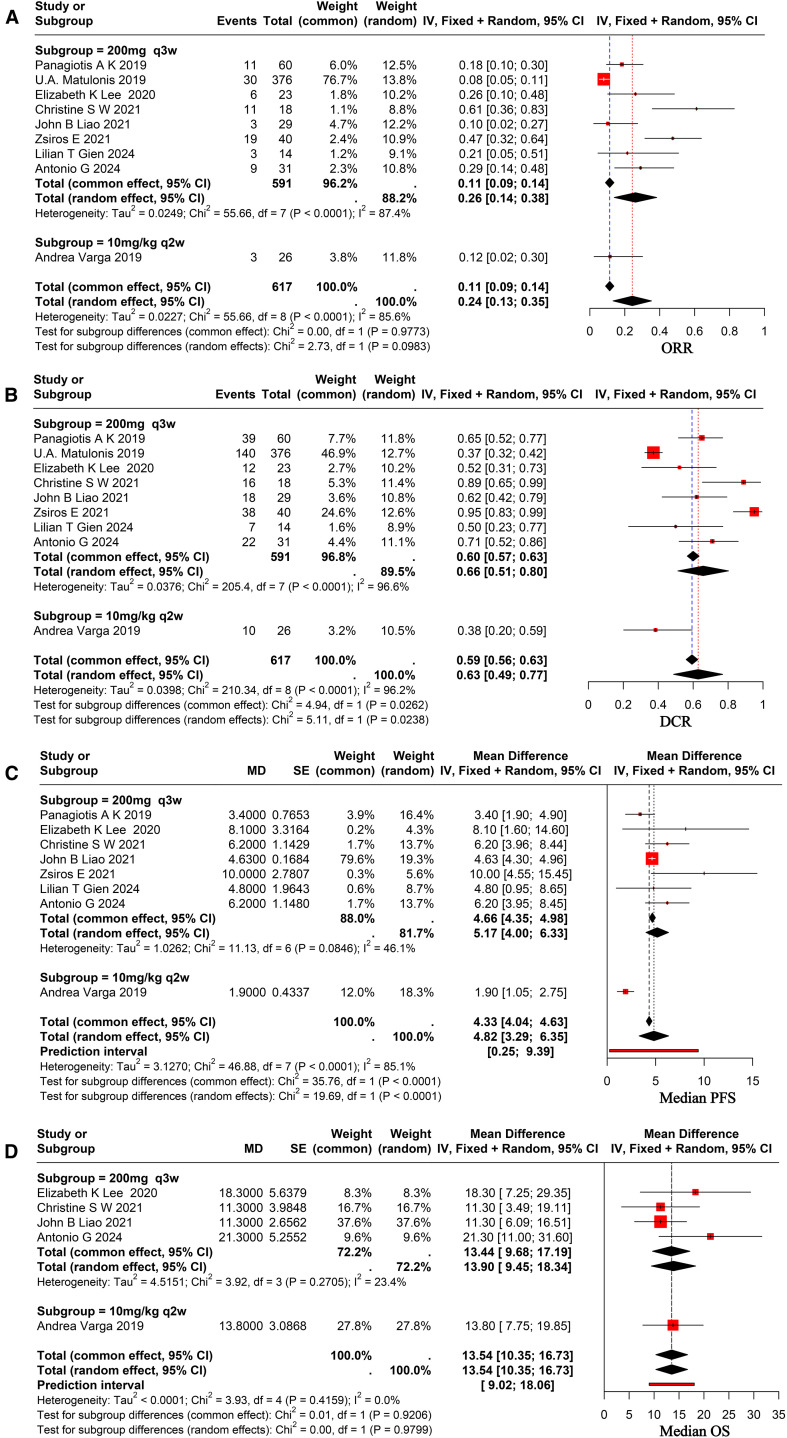
Forest plot for pooled results of ORR **(A)**, DCR **(B)**, mPFS **(C)**, and mOS **(D)** in ovarian cancer patients receiving pembrolizumab.

All nine selected studies reported on the outcomes of ORR and DCR, with ORR varying from 7.98% to 61.11% and DCR from 37.23% to 95.00%. The analysis demonstrated a combined ORR of 24% (95% CI, 0.13-0.35), with significant heterogeneity among studies (I² = 85.6%), as shown in **Figure 2A**. The combined DCR was 63% (95% CI, 0.49-0.77), exhibiting significant heterogeneity across studies (I² = 96.2%), as illustrated in **Figure 2B**.

### 3.5 Survival analysis

Eight of the nine studies that were chosen reported complete mPFS, which ranged from 1.9 to 10.0 months, and five of them had full mOS, which ranged from 11.3 to 21.3 months. Utilizing arandom-effects model revealed a pooled mPFS of 4.82 months (95% CI, 3.29–6.35), indicating significant heterogeneity across studies (I² = 85.1%), as shown in **Figure 2C**. The pooled mOS was 13.54 months (95% CI, 10.35–16.73), with no significant heterogeneity among studies (I² = 0.0%), as shown in **Figure 2D**.

### 3.6 Subgroup analysis

**Figure 3:**
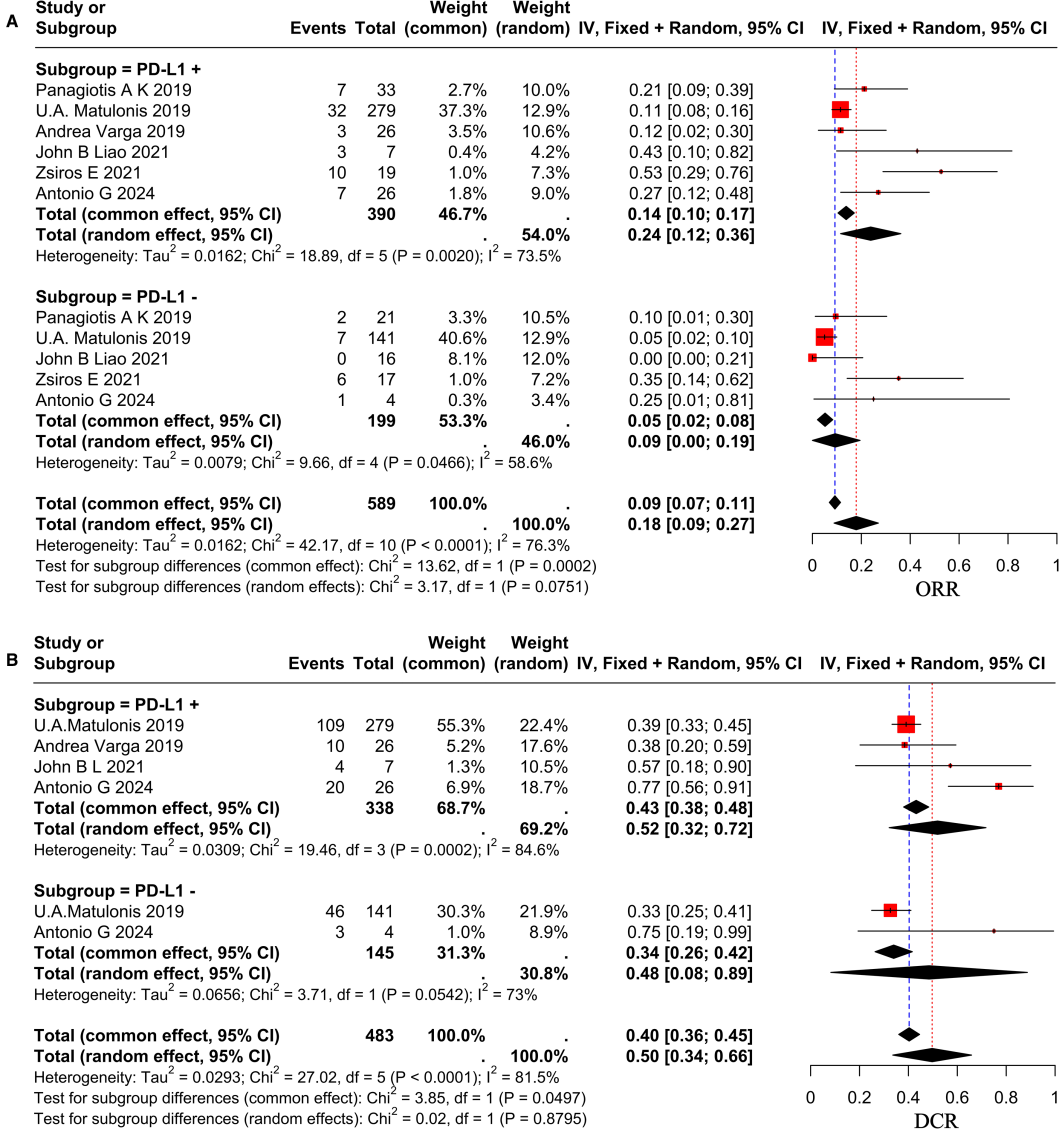
Forest plot showing subgroup analysis of ORR **(A)** and DCR **(B)** in different PD-L1 status group.

Following a comprehensive evaluation of all available studies, a subgroup analysis was conducted with patients based on their PD-L1 status and dosage. The study results demonstrated that the ORR for patients with PD-L1-positive status was 24% (95% CI, 0.12–0.36), while the ORR for patients with PD-L1-negative status was 18% (95% CI, 0.09–0.27), as illustrated in **Figure 3A**. It revealed no statistically significant differences between the subgroups (p = 0.07 > 0.05). The DCR was 52% (95% CI, 0.32–0.72) in PD-L1-positive patients and 48% (95% CI, 0.08–0.89) in PD-L1-negative patients, as demonstrated in **Figure 3B**. And it revealed no statistically significant differences (p = 0.879 > 0.05). To ascertain the therapeutic efficacy of different doses of pembrolizumab, a comparative analysis will be conducted utilizing various doses of the aforementioned drug. The ORR for patients receiving a dose of 200 mg every three weeks was 26% (95% CI, 0.13–0.33), while the ORR for the 10 mg/kg every two weeks (q2w) dose was 12% (95% CI, 0.02–0.30). The study revealed no statistically significant differences between the two groups (p = 0.09 > 0.05), as demonstrated in **Figure 2A**. The combined DCR for the former was 66% (95% CI, 0.51–0.80), in comparison to 38% (95% CI, 0.20–0.59) for the latter, with a significant discrepancy between the two (p = 0.02 < 0.05), as demonstrated in **Figure 2B**. The pooled mPFS for the former group was 5.17 months (95% CI, 4.00–6.33), in comparison with 1.90 months (95% CI, 1.05–2.75) for the latter group, with a significant difference between the two groups (p < 0.0001), as demonstrated in **Figure 2C**. The pooled mOS was 13.9 months (95% CI, 9.45–18.34) in the former group and 13.80 months (95% CI, 7.75–19.85) in the latter group, with no significant difference between the two groups (p=0.97 > 0.05), as demonstrated in **Figure 2D**. The classification criteria for patients’ tBRCA status, platinum-free treatment interval, platinum treatment status, and number of prior treatment lines were not suitable for subgroup analysis due to the large amount of missing data.

### 3.7 Adverse events

**Figure 4:**
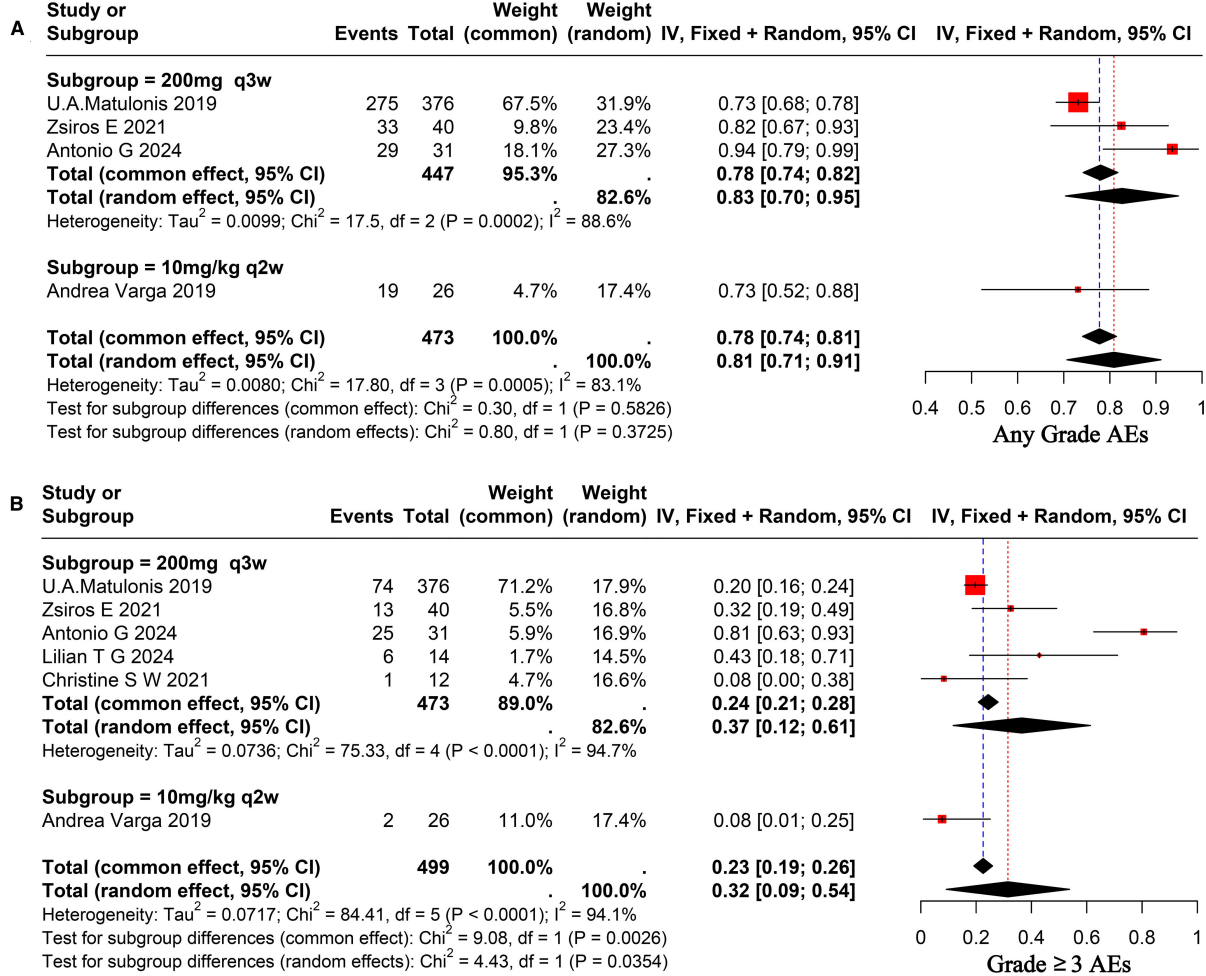
Forest plot for pooled results of any grade AEs **(A)**, ≥ grade 3AEs **(B)** on ovarian cancer patients receiving pembrolizumab.

A comprehensive review of the enrolled literature was conducted to collate the AEs experienced by OC patients during treatment. The majority of patients experienced grade 1–2 AEs, with an overall incidence rate of 81% (95% CI, 0.71–0.91) for AEs of any grade. The heterogeneity among studies was found to be significant (I² = 83.1%), as illustrated in **Figure 4A**. The incidence rate of grade three and above AEs was 32% (95% CI, 0.09–0.54), exhibiting significant heterogeneity among trials (I² = 94.1%), as seen in **Figure 4B**. A comparative analysis will be conducted to investigate the safety of various doses of pembrolizumab. The incidence rate of any grade AEs at a dose of 200 mg administered every three weeks was 83% (95% CI, 0.70–0.95), whereas the incidence rate for the 10 mg/kg administered every two weeks was 73% (95% CI, 0.52–0.88). No significant difference was observed between the two groups (p = 0.37 > 0.05), as illustrated in **Figure 4A**. The incidence rate of grade ≥3 AEs for the former was 37% (95% CI, 0.12–0.61), while for the latter it was 8% (95% CI, 0.01–0.25), indicating a significant difference between the two (p = 0.03 < 0.05), as illustrated in **Figure 4B**. The most AEs were fatigue (0.38), nausea (0.23), and fever (0.14), with a Grade 1–2 fatigue incidence rate of 38% (95% CI, 0.24–0.52). The incidence rate of grade ≥3 fatigue was much lower, recorded at 2% (95% CI, 0.01–0.04). The probability of encountering grade 1–2 nausea was 23% (95% CI, 0.11–0.34). The likelihood of encountering grade ≥3 nausea was markedly decreased to 1% (95% CI, 0.00–0.01). The probability of fever was 14% (95% CI, 0.04–0.23). The most common hematological toxicity is anemia, occurring at an incidence rate of 18% (95% CI, 0.01–0.35) (I² = 87.7%). The predominant immune-related AEs are Grade 1–2 hypothyroidism and Grade 1–2 hyperthyroidism, occurring at incidence rates of 17% (95% CI, 0.07–0.27) and 7% (95% CI, 0.04–0.09), respectively. The probability of grade ≥3 hypothyroidism and hyperthyroidism is significantly decreased. The results of the above AEs are shown in **Supplementary Figure 1**. A total of 32 patients discontinued treatment due to serious AEs, as reported in studies. The primary AEs resulting in discontinuation comprised skin toxicity, cholecystitis, diarrhea, and pulmonary embolism, among others. Three patients died due to serious AEs (31, 36).

### 3.8 Sensitivity analysis

The Leave-One-Out method is utilized to determine the influence of individual studies on overall results by analyzing the effect size and confidence interval of each study post-exclusion in relation to the overall combined effect size. Results indicated that the majority of results were not significantly affected by any individual study. The sensitivity analysis results are presented in **Supplementary Figure 2**.

### 3.9 Publication bias

This meta-analysis employed the Egger test to evaluate publication bias, indicating significant bias for mPFS and ORR (p < 0.05). The results were subsequently adjusted using the trim-and-trim method, as shown in **Supplementary Figure 3**. Publication bias is notably significant due to the lack of a control group in single-arm clinical trials, complicating the management of patient selection bias. Multiple factors, including the patient’s health status and treatment history, can affect patient selection (37, 38).

## 4 Discussion

Our systematic review and meta-analysis provides a comprehensive evaluation of pembrolizumab for advanced or recurrent OC, synthesizing evidence from multiple single-arm studies. By incorporating data from 9 studies involving 625 patients, our aggregated analysis demonstrates that pembrolizumab exhibits clinically meaningful efficacy, with an ORR of 24% and a DCR of 63%, alongside manageable safety. Specifically, the ORR signifies a notable antitumor response, while the DCR reflects disease stabilization in a substantial proportion of patients, suggesting therapeutic benefit. As for survival outcomes, the pooled mPFS was 4.82 months, and mOS reached 13.54 months, highlighting pembrolizumab potential to extend survival in this challenging population. The incidence of any grade AEs was 81%, with grade ≥3 AEs at 32%, supporting an acceptable safety profile consistent with prior reports of immune checkpoint inhibitors. This study offers pivotal evidence-based guidance for integrating pembrolizumab into treatment strategies for advanced or recurrent OC.

OC represents the most lethal gynecological malignancy, distinguished by its high malignancy, significant invasiveness, and elevated recurrence rate (39). Advanced and recurrent OC is associated with a poor prognosis, posing substantial treatment challenges and serving as a focal point for clinical research. The identification of effective treatment methods is a global common goal. Treatment options for OC currently encompass surgery, chemotherapy, and emerging modalities, including targeted therapy and immunotherapy. The category of targeted therapy drugs primarily encompasses anti-angiogenic agents, PARP inhibitors, and additional classes of drugs (40). Bevacizumab, an anti-angiogenic drug, was the inaugural pharmaceutical agent approved by the U.S. FDA and the European Medicines Agency (EMA) for first-line maintenance therapy in OC. The findings from GOG-0218 and ICON7 indicated substantial improvements in progression-free survival (41, 42). As research continues to advance, bevacizumab treatment for patients with platinum-resistant and platinum-sensitive recurrent OC has shown not only PFS benefits but also significant improvements in ORR (43, 44). The results of the PRIMA trial suggest that niraparib (a PARP inhibitor) can significantly prolong PFS in patients with advanced OC (45).

Immunotherapy has become an important treatment option after surgery, radiotherapy, chemotherapy, and targeted therapy, offering new strategies for managing advanced, recurrent, or metastatic malignant tumors (46). Pembrolizumab, the inaugural PD-1 inhibitor approved in the United States, has ushered in a novel phase of tumor immunotherapy. KEYNOTE-028 represented the inaugural clinical trial utilizing pembrolizumab monotherapy for the treatment of patients with PD-L1-positive advanced OC. Twenty-six patients underwent treatment, resulting in an ORR of 11.5%, a DCR of 62%, a mPFS of 1.9 months, and a mOS of 13.8 months (47). A clinical trial testing pembrolizumab with niraparib for patients with recurrent platinum-resistant OC (KEYNOTE-162) found that, out of 60 patients who could be evaluated, the ORR was 18%, and the DCR was 65%, with three patients having complete responses. The mPFS was 3.4 months, while the mOS was not reached (31). A study examining the combination of pembrolizumab and carboplatin for recurrent platinum-resistant OC indicated that out of 29 patients, the ORR was 10.3%, the DCR was 62%, the mPFS was 4.63 months, and the mOS was 11.3 months (35). A separate study assessing the efficacy of pembrolizumab in conjunction with bevacizumab and oral cyclophosphamide for recurrent OC indicated an ORR of 47.5%, a DCR of 95%, a mPFS of 3.5 months, and a mOS of 85 months (48). Based on the data presented, it can be found that pembrolizumab is effective in the treatment of advanced or recurrent OC, with its efficacy further improved when used in conjunction with other treatment regimens. The findings of this meta-analysis substantiate the effectiveness of pembrolizumab in the treatment of advanced or recurrent OC.

This study performed subgroup analysis only based on varying PD-L1 statuses and doses, owing to the absence of standardized subgroup classification criteria across studies. The findings indicated that of the 9 studies incorporated in this analysis, 1 study did not assess patients for PD-L1 status (49), 2 studies evaluated a total of 42 patients for PD-L1 status without conducting subgroup analysis based on this status (33, 34), while 6 studies identified 390 PD-L1-positive patients and 199 PD-L1-negative patients, performing subgroup analyses based on PD-L1 status. The ORR in the PD-L1-positive group was 24%, which was marginally higher than the 18% observed in the PD-L1-negative group. However, this difference was not statistically significant (p=0.07>0.05). A systematic meta-analysis examining the efficacy of PD-1 or PD-L1 inhibitors in cancer treatment in relation to PD-L1 expression status demonstrated that treatment outcomes in the PD-L1-positive group were superior to those in the PD-L1-negative group, with the difference being statistically significant (47). This study did not find any significant differences in efficacy between the two groups. The reason for this may be that the data related to PD-L1 status in this study was systematically missing, leading to inconsistent results with other studies. It may also be due to the single-arm clinical trial or the limited number of patients enrolled. Similarly, just as 7T fMRI with T2prep BOLD sequences can detect subtle functional changes in the olfactory bulb and piriform cortex in Parkinson’s disease patients (50), OC research could use more sensitive detection methods to quantify PD-L1 expression. And just as EEG can reveal neurobiological differences between different symptom subtypes of ADHD (51), OC studies could explore multimodal biomarkers to optimize patient stratification. Nevertheless, the implementation of these methods is currently challenging. Furthermore, the implementation of additional high-quality randomized controlled trials is recommended to verify the difference in efficacy between the two groups.

Subgroup analysis results based on different dosing regimens showed that the subgroup receiving 200 mg q3w demonstrated significantly superior outcomes in terms of ORR and mPFS compared to the subgroup receiving 10 mg/kg q2w (p < 0.05). However, no statistically significant difference was observed between the two groups in terms of DCR and mOS (p > 0.05). With regard to the safety assessment, no statistically significant differences were observed in the probability of any grade AEs occurring between the two groups (p > 0.05). However, the probability of occurrence of ≥3-grade AEs was significantly lower in the subgroup receiving a dose of 10 mg/kg q2w compared to the subgroup receiving a dose of 200 mg q3w. The findings indicate that, regarding short-term efficacy, the group administered a dose of 200 mg q3s may outperform the group receiving a dose of 10 mg/kg q2w. In terms of long-term efficacy, no significant difference was observed between the two groups. In the context of severe AEs, individualized dosing may be more advantageous than standardized dosing. Further high-quality RCTs are necessary to validate these findings.

During the course of immunotherapy, ICIs exert their antitumor immunotherapeutic effects by regulating T cell activity. In addition to their direct action against tumor cells, these agents have the potential to induce systemic immune-related adverse events (irAEs) (52). Conventional chemotherapy or radiotherapy has been shown to cause tissue damage that is related to the site of action. The resulting AEs are both fixed and predictable. Conversely, the AEs associated with immunotherapy are distinct, with irAEs being both widespread and unpredictable (53). irAEs can potentially affect all organs and systems in the human body. AEs involving the skin, gastrointestinal tract, endocrine system, respiratory system, and musculoskeletal system are relatively common, as is thyroid dysfunction. However, AEs involving the cardiovascular and pulmonary systems are relatively less common (54). irAEs have been observed to occur within a time frame ranging from a few weeks to several months following the initiation of therapy, with the potential for occurrence after the cessation of treatment also being documented (54). irAEs vary from asymptomatic to severe or life-threatening and are categorized into five grades, from grade 1 to grade 5. Certain immune-related AEs, such as hypothyroidism, may be managed with hormone replacement therapy, eliminating the necessity for corticosteroids (55). Grade 1–2 irAEs are typically addressed through symptomatic treatment, which may involve the use of topical or oral corticosteroids. IrAEs that impact the heart, lungs, liver, or nervous system are more severe and necessitate high-dose intravenous corticosteroids as the initial treatment approach. Grade 3–4 irAEs frequently necessitate hospitalization and are predominantly managed with systemic corticosteroids, either orally or intravenously (56).

The study revealed that the incidence rate of AEs of any grade was 81% in the combined treatment group, which is higher than the 73% observed in the pembrolizumab monotherapy group (47). The high number of AEs is mainly because most studies used combination therapy that included pembrolizumab, like treatments with carboplatin, cyclophosphamide, and bevacizumab. The AEs induced by these drugs during treatment are challenging to differentiate from the irAEs caused by pembrolizumab. Consequently, the incidence of common AEs in the entire study population was relatively high, including fatigue, nausea, vomiting, fever, and hypertension. But pembrolizumab may not be the main cause of these AEs, implying its safety profile may be better than this study’s. The most prevalent irAEs observed in this study were grade 1–2 hypothyroidism and grade 1–2 hyperthyroidism, with incidence rates of 17% and 7%, respectively. The probability of grade ≥3 hypothyroidism and hyperthyroidism was found to be significantly reduced. In the KEYNOTE-100 study, two patient deaths were attributed to AEs related to pembrolizumab treatment, including one case of Stevens-Johnson syndrome and one case of hypoaldosteronism (31). In the LEAP-005 study, one patient died due to treatment-related AEs, specifically hypovolemic shock. We outline the sequence of events that transpired below: Initially, the patient exhibited gastrointestinal symptoms, including nausea and emesis, which were reminiscent of coffee grounds. These symptoms progressed to renal failure, accompanied by lactic acidosis, ultimately resulting in death due to multi-organ failure. After a detailed review by the investigation team, it was found that this death was linked to the use of lenvatinib along with pembrolizumab (36).

The age range of patients included in this meta-analysis was broad (25–89 years), with the median age in each study concentrated between 55 and 65 years. The lack of detailed data from each study precluded subgroup analyses by age, thereby limiting our ability to assess the influence of age on the efficacy and safety of immunotherapy for OC. As the age of the people increases, factors such as chronic viral stimulation, the senescence-associated secretory pattern (SASP) of aging cells, and abnormal immune training collectively mediate inflammatory aging, which is a key risk factor for morbidity and mortality in the elderly (57). The impact of age on the efficacy and safety of tumor immunotherapy is a complex and multifaceted area of research. As demonstrated in extant literature and evidenced by existing research, the impact of age on the efficacy and safety of immunotherapy remains a contentious and complex issue. The two studies included in this meta-analysis with the greatest discrepancy in median age, i.e., Christine’s study (median age 55 years) (34)and John B Liao’s study (median age 65 years) (35), demonstrated no significant difference in mOS results, which is consistent with the results of the EMPOWER-Lung 3 trial (58). However, Jaclyn Sceney’s study yielded contrary results, suggesting that advancing age may reduce the efficacy of immunotherapy (59). Current research findings indicate variability in the impact of age on the safety of immunotherapy. A cohort study conducted by Nebhan CA et al. on monotherapy with immune checkpoint inhibitors in patients aged 80 years and older found no significant difference in the incidence of irAEs among the three groups: patients aged less than 85 years, those aged 85–89 years, and individuals aged 90 years and older (60). Research indicates that the occurrence of severe irAEs in patients aged 80 years and older does not show a significant increase; however, the associated mortality risk from these adverse reactions warrants careful consideration (61).

This meta-analysis, based entirely on single-arm studies, significantly increased the levels of heterogeneity and publication bias in this research. A single-arm trial constitutes a clinical trial design in which a single experimental group is established, devoid of a parallel control group. The study primarily compares results with external controls, such as historical data or target values, and does not adhere to the principles of randomization and blinding (62). Single-arm trials are predominantly utilized in the initial phases of drug development, especially during the exploratory stage of efficacy evaluation for cancer and rare diseases. Recently, there has been a rise in the application of single-arm trials in clinical settings, with several high-quality results being integrated into clinical guidelines or utilized as evidence for drug approval (62, 63). Single-arm clinical trials possess specific limitations. The lack of a control group limits the interpretation of results to the intervention measures, consequently diminishing the reliability of the evidence. Comparing with external historical data is the sole option due to the absence of parallel controls. The variations among these studies complicate evaluation and may introduce bias, thereby impacting the accuracy of efficacy and safety assessments (64). Thus, there are certain restrictions on this meta-analysis, which is based on single-arm clinical studies. Moreover, the study predominantly comprised patients from middle- and high-income countries, including the United States, China, and France. However, data from regions such as Africa and Latin America are lacking, which has a consequential effect on the representativeness of the global landscape of patients with advanced/recurrent OC. The present study documented a mOS of 13.54 months. However, it should be noted that only five studies furnished complete mOS data, and the longest follow-up duration did not surpass 21.3 months. However, there is a paucity of long-term survival data, with a maximum follow-up period of two years. Moreover, pivotal clinical concerns, such as the mechanisms of resistance to pembrolizumab and the modifications to treatment protocols following such resistance, remained unaddressed, impeding the provision of guidance for long-term treatment strategies. It is evident that further clinical trials and fundamental research are required in a range of regions in order to investigate the efficacy and safety of pembrolizumab in the treatment of OC.

Due to the limitations of this study, future large-scale prospective RCTs are necessary to validate the efficacy and safety of pembrolizumab for treating advanced or recurrent OC. The differences in efficacy among various combination therapy regimens and subgroups categorized by distinct criteria warrant further exploration. The drug’s mechanism of action and the factors contributing to AEs necessitate further investigation to improve the efficacy and safety of pembrolizumab in treating advanced or recurrent OC.

## 5 Conclusion

In summary, this meta-analysis demonstrates that treatment with pembrolizumab for advanced or recurrent OC significantly improves objective ORR and DCR and prolongs survival. Despite the occurrence of treatment-related AEs in a proportion of patients, these events are generally manageable. However, due to the limited clinical data available, the need for large-scale, multicenter, prospective RCTs in the future is evident, with the objective of validating the efficacy and safety of the treatment.

## Data Availability

The original contributions presented in the study are included in the article/**Supplementary Material**. Further inquiries can be directed to the corresponding author.
