# Supplementary material for: Efficacy and safety of pembrolizumab for the treatment of advanced or recurrent ovarian cancer: a meta-analysis based on single-arm studies
Source: Front Immunol. 2025 Oct 1;16:1662455. doi: 10.3389/fimmu.2025.1662455 (PMC12521426; doi:10.3389/fimmu.2025.1662455)
Supplement: Supplementary file 1 [file DataSheet1.docx]

Supplementary Material

# Supplementary Data

None.

# Supplementary Figures and Tables

## Supplementary Figures


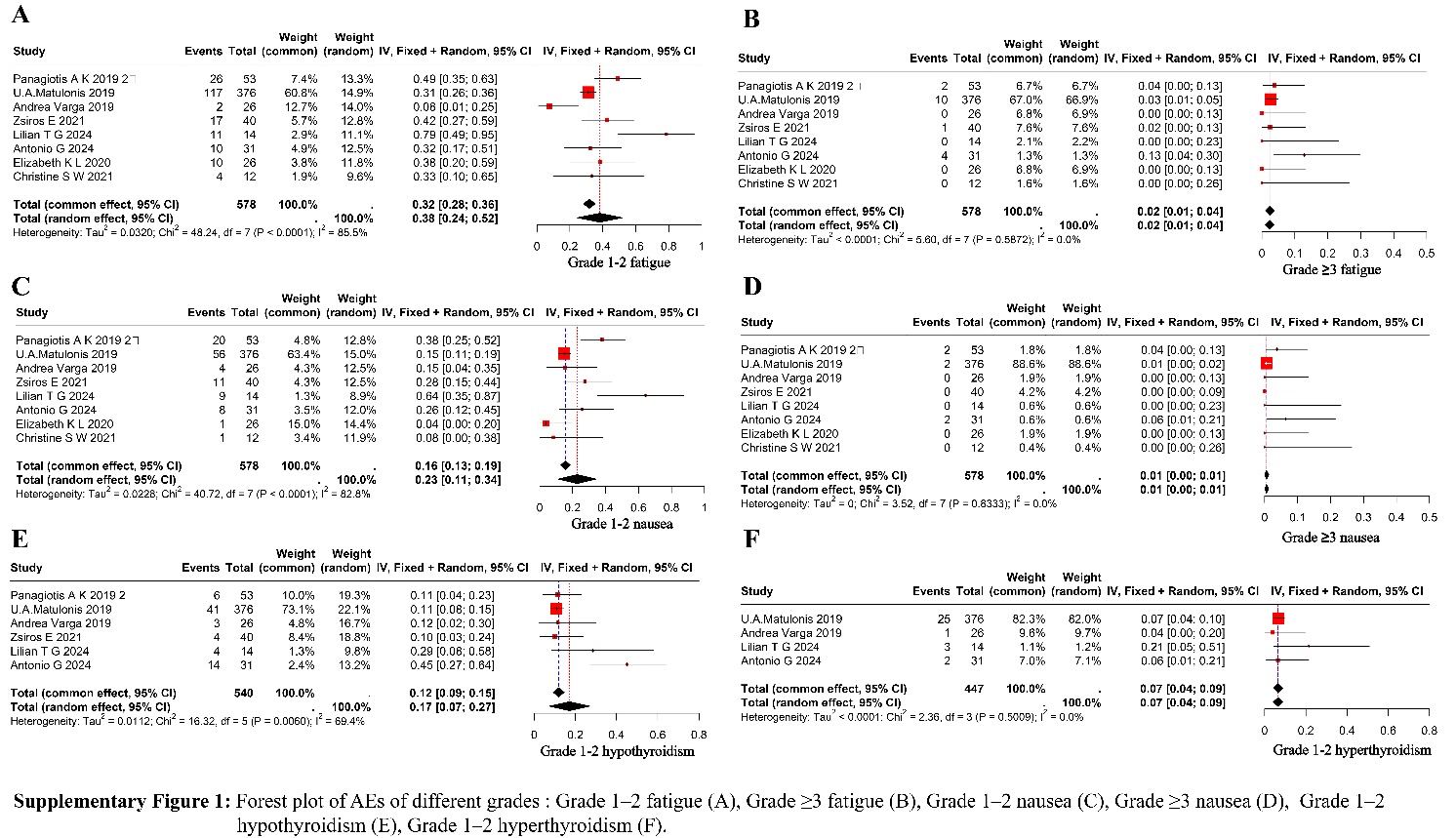


**Supplementary Figure 1.** Forest plot of AEs of different grades: Grade 1-2 fatigue (A), Grade 23 fatigue(B), Grade 1-2 nausea (C), Grade 23 nausea (D), Grade 1-2 hypothyroidism (E), Grade 1-2 hyperthyroidism (F).


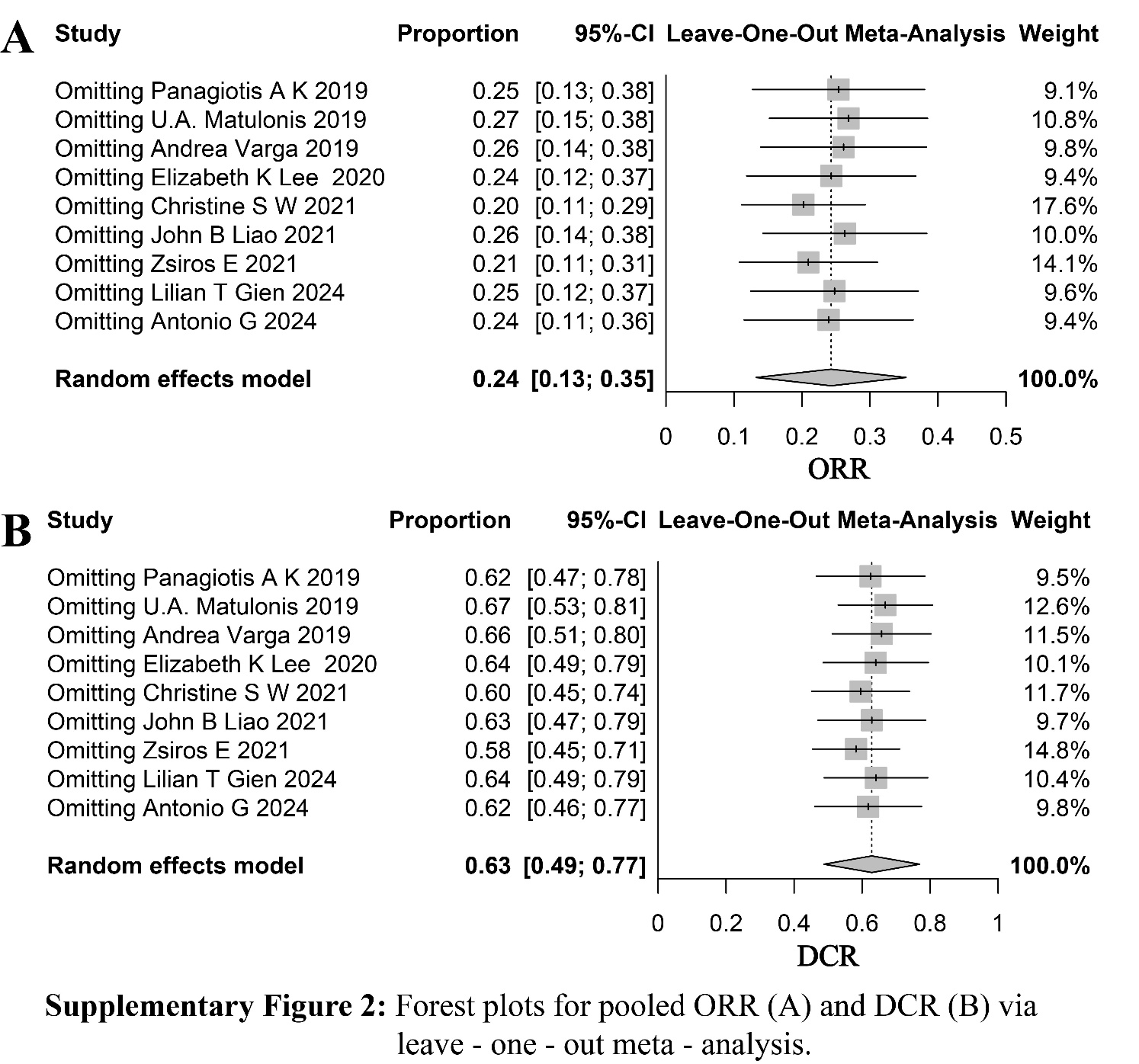


**Supplementary Figure 2.** Forest plots for pooled ORR (A) and DCR (B) via leave-one-out meta-analysis.


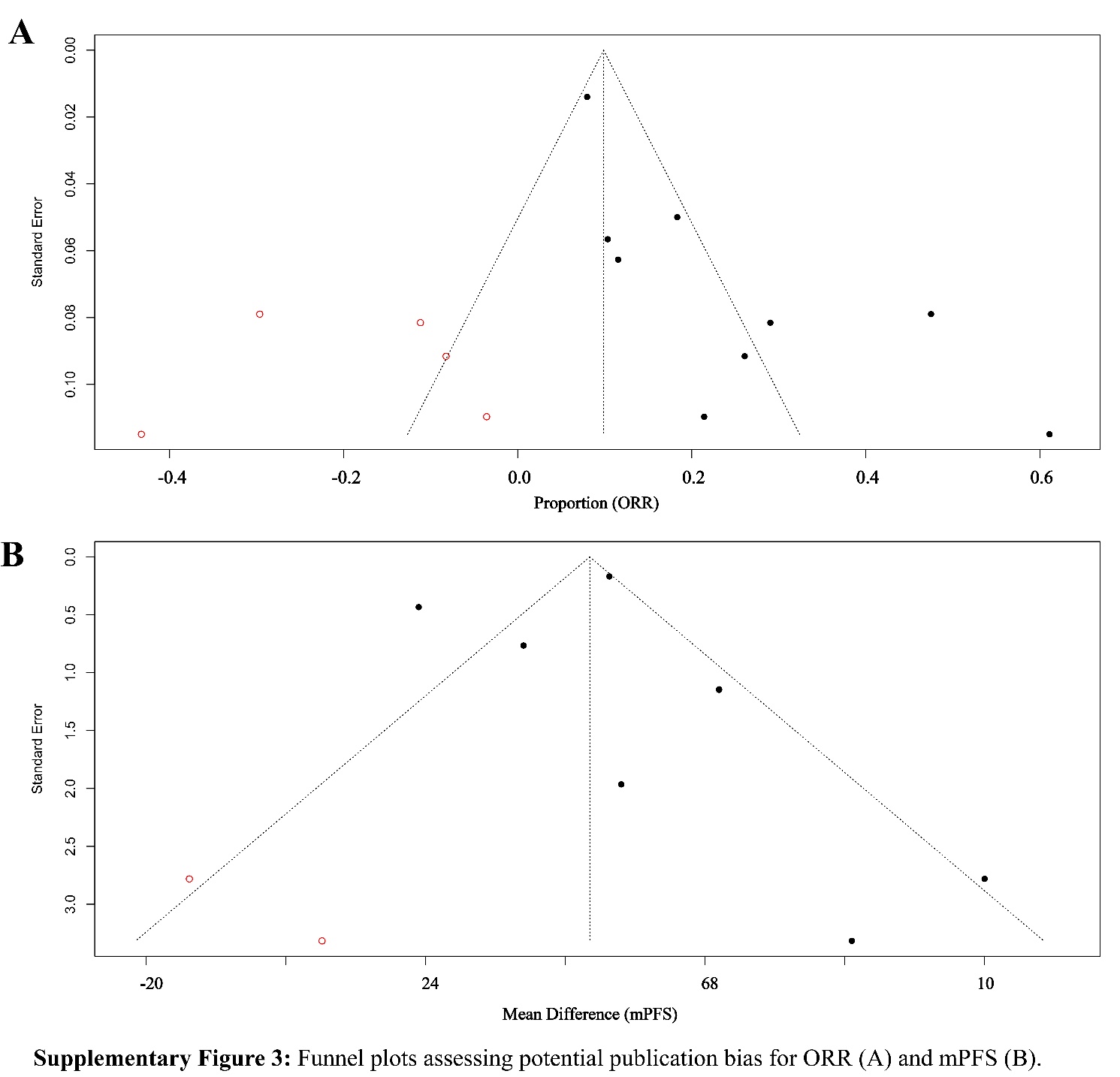


**Supplementary Figure 3.** Funnel plot (reduction-addition analysis) of ORR(A), mPFS(B) .
